# Daily Social Isolation Maps Onto Distinctive Features of Anhedonic Behavior: A Combined Ecological and Computational Investigation

**DOI:** 10.1016/j.bpsgos.2024.100369

**Published:** 2024-07-31

**Authors:** Valeria Gigli, Paola Castellano, Valerio Ghezzi, Yuen-Siang Ang, Martino Schettino, Diego A. Pizzagalli, Cristina Ottaviani

**Affiliations:** a Department of Psychology, Sapienza University of Rome, Rome, Italy; b Department of Experimental, Diagnostic and Specialty Medicine, University of Bologna, Bologna, Italy; c Department of Social and Cognitive Computing, Institute of High Performance Computing, Agency for Science, Technology and Research, Singapore; d IRCSS Istituto delle Scienze Neurologiche di Bologna, Bologna, Italy; e Center for Depression, Anxiety and Stress Research, McLean Hospital, Belmont, Massachusetts; f Department of Psychiatry, Harvard Medical School, Boston, Massachusetts; g Neuroimaging Laboratory, IRCCS, Santa Lucia Foundation, Rome, Italy

**Keywords:** Anhedonia, Ecologic momentary assessment, Probabilistic reward task, Reward learning, Reward sensitivity, Social isolation

## Abstract

**Background:**

Loneliness and social isolation have detrimental consequences for mental health and act as vulnerability factors for the development of depressive symptoms, such as anhedonia. The mitigation strategies used to contain COVID-19, such as social distancing and lockdowns, allowed us to investigate putative associations between daily objective and perceived social isolation and anhedonic-like behavior.

**Methods:**

Reward-related functioning was objectively assessed using the Probabilistic Reward Task. A total of 114 unselected healthy individuals (71% female) underwent both a laboratory and an ecological momentary assessment. Computational modeling was applied to performance on the Probabilistic Reward Task to disentangle reward sensitivity and learning rate.

**Results:**

Findings revealed that objective, but not subjective, daily social interactions were associated with motivational behavior. Specifically, higher social isolation (less time spent with others) was associated with higher responsivity to rewarding stimuli and a reduced influence of a given reward on successive behavioral choices.

**Conclusions:**

Overall, the current results broaden our knowledge of the potential pathways that link (COVID-19–related) social isolation to altered motivational functioning.

## Plain Language Summary

Loneliness and social isolation negatively impact mental health and contribute to depressive symptoms like anhedonia. With COVID-19 restrictions such as social distancing, we examined how daily social isolation, measured ecologically, is related to anhedonic behavior. We tested 114 healthy adults using a task that measured their responses to rewards. Greater isolation was linked to an increased response to rewards but also to a reduced ability to learn from them, which lessened the influence of rewards on future behavior. These findings highlight potential mechanisms that link social isolation to changes in motivation, ultimately leading to depressive symptoms.

The consequences of social isolation for symptoms and maladaptive behaviors have been widely investigated in clinical and preclinical studies. For example, it is well established that chronic social isolation elicits anhedonia (operationalized as a reduction in sucrose intake and sucrose preference) in rodents (1, 2, 3). Converging evidence from human investigations points to the bidimensionality of the construct: on the one hand, social isolation (or social disconnectedness) has been defined in terms of a restricted network of relationships and objective lack of interaction with others or participation in social activities (4,5). On the other hand, emotional isolation (5), also called perceived isolation (6), has been conceptualized as the subjective perception of lack of companionship, intimacy, and support and seems to be more strictly associated with feelings of loneliness, a sense of dissatisfaction with one’s connections, and qualitative, rather than quantitative, social deficiencies (7). Importantly, studies of humans suggest that both dimensions of social isolation and perceived isolation predict anhedonic symptoms, depression, anxiety (8,9), poorer mental health (10), and morbidity more generally (11). Conversely, a higher level of social support is a protective factor for anhedonia (12), suicide, and suicide risk (13) and is associated with a better quality of life and reduced functional decline in individuals with depression (14).

Most longitudinal studies that have attempted to shed light on causal relationships between loneliness and depressive symptoms have pointed to the perception of social isolation, loss of significant others, and a poor interpersonal network as risk factors for the development of depression (15, 16, 17), while others have described reciprocal influences between the 2 constructs (8,18).

The reviewed findings are consistent with the assumption of the social baseline theory, according to which the social network is the primary source of safety (19) and of the social safety theory, which further hypothesizes that social isolation and rejection upregulate inflammatory activity mainly via bidirectional neural-immune communication, ultimately increasing all-cause mortality (20,21).

The current study was conducted during a major period of social deprivation amid strict COVID-19 measures. Even following the conclusion of national lockdowns, Italy (including the area in which the current study was performed) continued to face prolonged restrictions at the regional level until early 2022, namely constraints on the number of individuals permitted in public or private spaces, implementation of curfews, and the transition to online learning and remote work. This situation afforded us the opportunity to investigate the effects of varying degrees of loneliness and isolation in a naturalistic setting.

The aim of the current study was to investigate whether (objective or perceived) social isolation would be associated with anhedonic symptoms in an unselected healthy sample. Importantly, an ecological momentary assessment (EMA) was implemented to provide a daily estimation of the number and duration of (i.e., objective social isolation) as well as the desire and need for (i.e., perceived isolation) social interactions. Moreover, assessment of anhedonic symptoms did not rely on self-reports but instead was evaluated based on participants’ performance on the Probabilistic Reward Task (PRT) (22), in which anhedonic behavior is operationalized as the ability to modify behavior as a function of rewards. Overall, we hypothesized positive associations between objective and subjective measures of social isolation and anhedonic behavior. Although it has been historically conceptualized as loss of pleasure (23), anhedonia is a complex and multifaceted symptom that may arise from impairments in several aspects of reward processing, including blunted reward learning or reward sensitivity (24,25). To parse the specific associations of each of these components with social isolation, computational modeling of trial-level performance on the PRT was implemented (26). Given the absence of previous investigations of this issue, we did not have a specific hypothesis about which of the 2 components would be more affected by social isolation.

Numerous studies have explored the relationship between objective or perceived social isolation and their associations with the multifaceted construct of anhedonia. However, the current study stands out by aiming to investigate this relationship not only through ecological assessment but also through a behavioral task. By dissecting behavioral performance into 2 components, we aimed at a closer understanding of which aspects of motivational functioning are more involved in this relationship. This approach holds promise to provide a more nuanced understanding of the mechanisms that underlie the interplay between social isolation and anhedonia.

## Methods and Materials

### Participants

This study is part of a larger research project (27), although the sample is not overlapping. Participants were recruited from among university students and the general population from May 2021 to November 2021. The protocol was approved by the local institutional review board (Protocol No. 1170/2021). The final sample included 114 participants (81 female, mean age 22.24 ± 2.93 years, range 20–30 years) (see Supplemental Section S1 for exclusionary criteria).

### Procedure

After signing the informed consent form, participants were asked to complete a set of online questions assessing sociodemographic, lifestyle, and medical information (e.g., age, weight, nicotine and alcohol consumption, physical activity) and a validated questionnaire to evaluate self-reported loneliness. Next, a laboratory visit was scheduled during which participants were asked to perform the PRT. Participants then received a full explanation of the EMA procedure and were asked to keep electronic diaries on their smartphones for 4 consecutive days, after which they were debriefed and compensated with the money that they won during the PRT.

### Assessment of Perceived Social Isolation

The Italian version of the Revised UCLA Loneliness Scale (28,29) was administered; it is a 20-item questionnaire that assesses the subjective experience of loneliness and subjective feelings of social isolation (detailed in Supplemental Section S2). Internal consistency in the current study was α = 0.867. Questions regarding the specific COVID-19 situation were also administered (see Supplemental Section S3 for the full list).

### Probabilistic Reward Task

The PRT (22) is a well-validated signal detection task recommended by the Research Domain Criteria (30) as a probe of participants’ ability to modify their behavior as a function of reward, thus providing an objective measure of reward responsiveness and reward learning (detailed in Supplemental Section S4 and Figure S1) (31).

Performance was analyzed with respect to response bias (log b), an empirically derived measure of systematic preference to choose the more frequently rewarded stimulus (rich). ΔResponse bias was computed as the systematic preference to identify the more frequently rewarded stimulus (response bias) during block 3 minus response bias during block 1 (22). To evaluate overall task performance, we also examined discriminability, which is participants’ ability to distinguish between the 2 stimuli reflecting task difficulty, as well as accuracy and reaction times (see Supplemental Section S4 for formulas and secondary analyses).

#### Computation Modeling of the PRT

To parse specific components of reward processing, the participants’ trial-level PRT data were fitted with 4 reinforcement learning models based on previously established methods (detailed in Supplemental Section S5) (26). Group priors in these models were computed via expectation maximization (32), and subject-specific parameters were estimated through Laplace approximation of the posterior distributions. Integrated group-level Bayesian information criterion factors were used to compare the model fits. The most parsimonious account of the data was provided by the action model, with a group-level log Bayes factor compared with the second-best belief model of 68 (which represents very strong evidence). This approach allowed for the derivation of 2 main parameters: 1) reward sensitivity, with higher scores denoting that a participant is more sensitive to rewards (mean = 2.02, SD = 0.35), and 2) learning rate (mean = −5.35, SD = 1.9), which measured the ability to learn from reward feedback, with higher scores indicating a stronger effect of reward on successive behavior.

In accordance with Huys *et al.*, parameters were computed in the transformed space to prevent issues with non-Gaussianity (see Supplemental Section S6 for details) (26).

### Ecological Momentary Assessment

The EMA was the last step of the research protocol and involved repeated sampling of individuals’ current experiences in real time and in natural environments with the purpose of minimizing recall bias and maximizing ecological validity (33).

Participants received preprogrammed emails containing a link to their electronic diary on Qualtrics.com. These emails were sent randomly within a 2-hour time window from the first Wednesday after the laboratory session until the following Saturday. On each of the 4 days, participants received 6 surveys, which were randomly distributed throughout their self-reported waking period and began at least 30 minutes after they woke up. The EMA questions were only available for 25 minutes after the initial notification. Answers were provided on a visual analog scale, and each diary took 1 to 2 minutes to be filled out. Based on previous studies (34), ad hoc questions were used to assess the quantity (“Since the last electronic diary, did you have any social interaction?”), duration (“How long did this interaction last?”), subjective perception (“How do you rate the valence of such interaction?”), and desire or need for social interactions (“Since the last electronic diary, have you missed interacting with people?” or “How hard have you desired to interact with someone?”). Participants had to choose “yes” or “no” for the question about the presence of interaction while the other answers were provided on a visual analog scale ranging from 1) 0 = “completely negative” to 10 = “completely positive” for valence; 2) 1 minute to 120 minutes or more for duration; and 3) 0 = “not at all” to 10 = “very much” for desire or need.

### Data Analysis

First, assumptions of normality, linearity, homogeneity of variances, and sphericity were checked. Then, the influence of potential confounders, such as age, sex, alcohol, and smoking, on the main variables of interest was assessed using correlational and *t* test analyses. The variables that yielded significant results in this preliminary check were included as covariates in all subsequent analyses.

Regarding PRT performance, 4 separate general linear models were performed. First, the general linear models were computed for response bias and discriminability as outcomes and block (block 1, block 2, block 3) as the within-participant variable. For accuracy and reaction time, stimulus (lean vs. rich) was included as an additional factor. Behavioral results regarding performance on the PRT have been reported elsewhere (27) and will not be detailed here (see Figure S1 for a graphical representation).

For the EMA component, a multilevel model was carried out with Mplus version 8.7 software (35) using robust maximum likelihood estimators with a full information maximum likelihood approach under the missing-at-random assumptions to handle missing data. Participants with <30% of valid assessments on the EMA measures were not retained for this analysis as they were in previous EMA studies in the context of anhedonia and motivational behavior (36,37).

The models were hypothesis driven and informed by the previously reviewed rodent and human findings on the detrimental effects of social isolation on anhedonic symptoms. A mediational chain was specified at the between-participants level: trait loneliness predicting valence of social interaction, which in turn predicted the duration of daily social interaction, which in turn predicted Δresponse bias (model 1) and the 2 components of the PRT (model 2). Duration of daily social interaction was also partialled out for trait loneliness, and trait loneliness was specified as being associated with the 2 components of the PRT. In model 2, the computational-based components of the PRT (i.e., reward sensitivity and learning rate) were treated as separate dependent between-participants variables within the overall nomological network (see Supplemental Section S8 for details on the models).

## Results

No correlations or differences emerged in the main variables of the study with respect to age, sex, or nicotine and alcohol consumption, with the exception of Δresponse bias, which was significantly higher in nonsmokers than smokers (*t*_112_ = 2.06, *p* = .02). Thus, general linear models were performed controlling only for smoking status also given previous findings of significant effects of nicotine craving on response bias (38).

The sample included 5 participants who lived alone, 7 who lived with a partner, 23 who lived with roommate(s), and 79 who lived with their family. Regarding consequences of COVID-19, 40 participants reported that they had undergone quarantine measures during the 6 months prior to the start of the study. Most participants (*n* = 81) highlighted a negative influence of COVID-19 restrictions, and 67 participants reported perceived social isolation. The average Revised UCLA Loneliness Scale score was 38.34 ± 8.37 (normative scores are 37.06 ± 10.91 for men and 36.06 ± 10.11 for women) (28).

**Figure 1:**
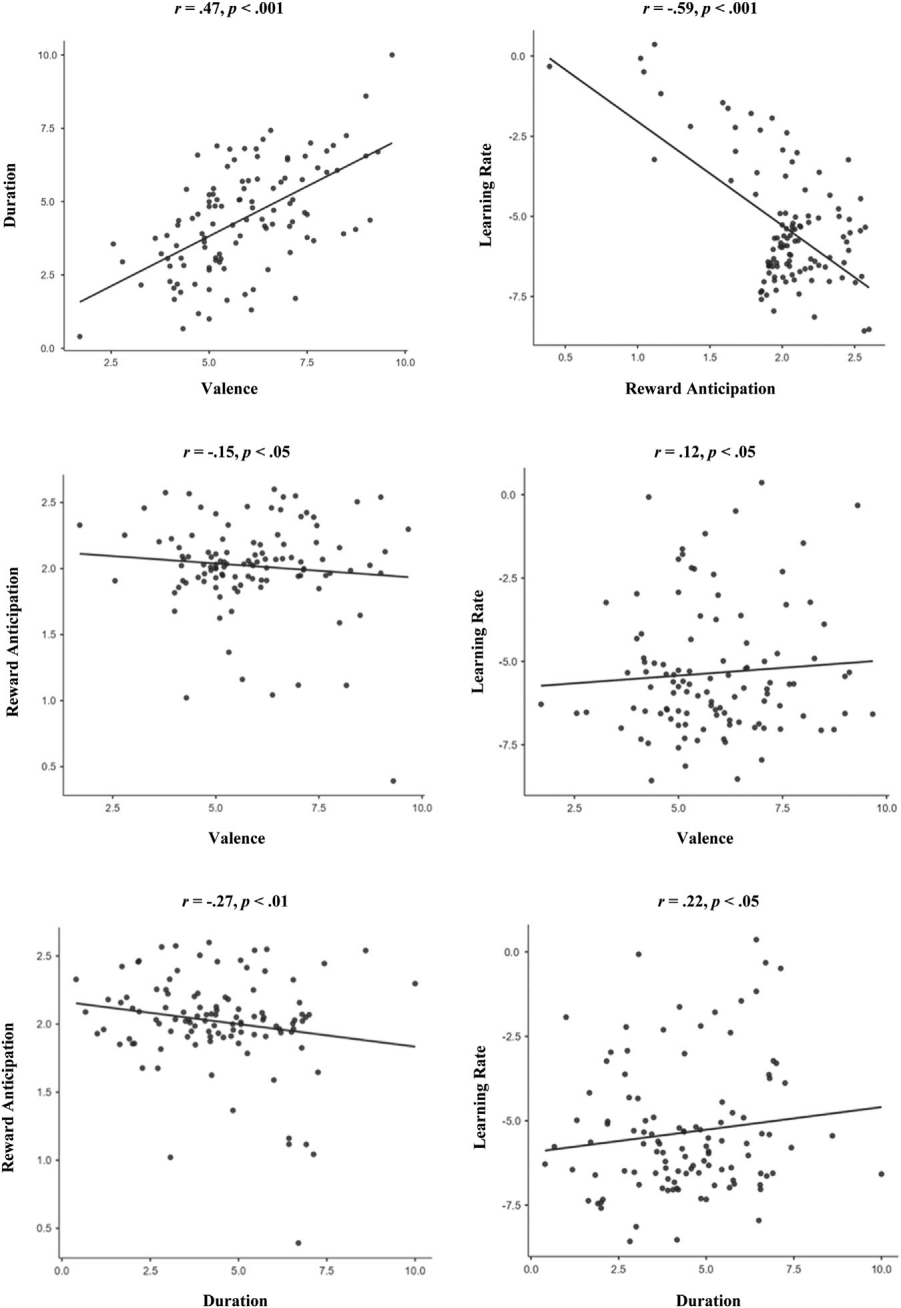
Scatterplots of the between-level associations between learning rate (in transformed space) and reward sensitivity (in transformed space) and momentary ecological assessment of valence and duration of daily social interaction.

In general, after excluding 1.76 of outlier trials, an increase in response bias from the first block to the following blocks emerged together with a concomitant increase in accuracy and a decrease in reaction time for rich (vs. lean) trials from block 1 to block 2 and 3. As expected, no effects of discriminability emerged. Collectively, these patterns confirm that the PRT elicited the intended behavioral effects. Consistent with previous accounts (26), reward sensitivity and learning rate were inversely associated (*r* = −0.59, *p* < .001) (see Table 1, Figure 1, and Supplemental Section S7 for outliers).

**Table 1:** Descriptive Statistics and Zero-Order Correlations for the Ecological Momentary Assessment Model Variables (*n* = 106)

		Descriptive Statistics			Correlations					
		Mean (SD)	Skewness	Kurtosis	1	2	3	4	5	6
1	ΔResponse Bias	0.09 (0.28)	0.33	0.13	–	0.20^a^	0.30^a^	0.00	−0.02	−0.05
2	Learning Rate	−5.35 (1.9)	1.06	0.06	–	–	−0.59^b^	0.12^c^	0.22^a^	0.02
3	Reward Sensitivity	2.02 (0.35)	−1.57	4.73	–	–	–	−0.15^c^	−0.27^a^	−0.04
4	Valence	5.81 (3.59)	−0.53	−1.08	–	–	–	–	0.47^b^	−0.36^b^
5	Duration	4.31 (3.77)	0.35	−1.40	–	–	–	0.64^b^	–	−0.15
6	Loneliness	34.47 (0.81)	0.80	0.66	–	–	–	–	–	–

ΔResponse bias indicates response bias during block 3 minus response bias during block 1; learning rate (in transformed space) indicates the ability to learn from reward feedback; reward sensitivity (in transformed space) indicates the internal worth of an external reward; valence indicates the valence of social interaction; duration indicates the duration of social interaction (objective measure of social isolation); loneliness indicates self-reported levels of loneliness assessed by the Revised UCLA Loneliness Scale. Mean, SD, skewness, and kurtosis refer to the between-participants level (for the ecologic momentary assessment variables, these are calculated across all available measurement occasions). Correlations below the diagonal pertain to the within-participants level, and those above the diagonal pertain to the between-participants level.

a *p* < .01.

b *p* < .001.

c *p* < .05.

### EMA Models

Eight participants reported a very large proportion of missing data points on EMA variables (>70% of the total), and consequently, they were excluded from the analyses. Thus, the final sample for EMA models comprised 106 individuals, who did not differ from those excluded from the analyses on any sociodemographic or between-person variables. The final average proportion of valid data points for the EMA measures was 59.34% (SD = 15.04), with an average number of data entries (16.14, SD = 3.93, range: 9–24) that was comparable to that obtained in previous studies that have examined social interactions (39,40).

Descriptive statistics and scatterplots related to the EMA models are summarized in Table 1 and Figure 1. Model 1 failed to show significant prediction of Δresponse bias by valence or duration of daily social interactions (detailed in Figure S2).

**Figure 2:**
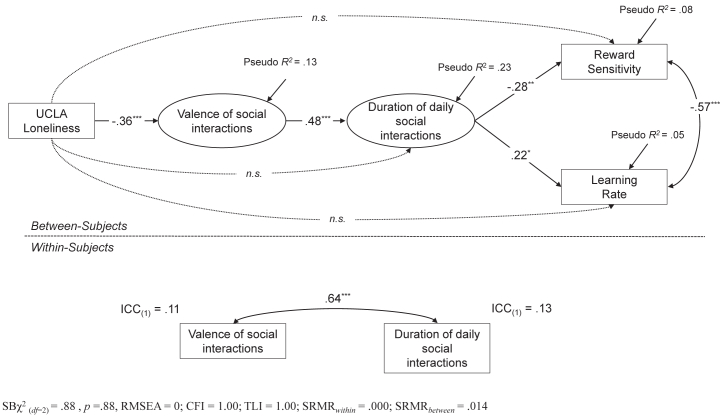
Completely standardized estimates from ecological momentary assessment (EMA) model 2. Dotted lines represent statistically nonsignificant paths. One-headed arrows represent direct effects, while double-headed arrows represent covariances. Squares indicated observed variables, while circles represent latent (between participants–level) components of EMA measures. ∗*p* < .05, ∗∗*p* < .01, ∗∗∗*p* < .001. CFI, comparative fit index; ICC, intraclass correlation coefficient; RMSEA, root mean square error of approximation; SB, Satorra-Bentler; SRMR, standardized root mean square residual; TLI, Tucker-Lewis index.

Estimates from the empirical EMA model 2 are reported in Figure 2. The model showed substantial fit to the data: Satorra-Bentler χ^2^_2_ = 0.88, *p* = .88. The unconditional intraclass correlation coefficients associated with the valence of social interactions and duration of daily social interactions largely varied across EMA occasions (approximately 90%), but a nonignorable component of their variability was associated with stable individual differences. In other words, scores on these variables varied to a consistent degree between assessments, although approximately 10% of their overall variability was explained by enduring individual characteristics. Trait loneliness significantly explained the stable component of the valence of social interactions stemming from EMA assessment (standardized β = −0.36, *p* < .001), which in turn significantly explained the stable component of the duration of daily social interactions (standardized β = 0.48, *p* < .001). Most importantly, the stable component of the duration of daily social interactions significantly explained both computational-based PRT parameters, exerting a negative effect on reward sensitivity (standardized β = −0.28, *p* < .01) and a positive effect on learning rate (standardized β = 0.22, *p* < .05). Overall, the nomological network explained 8% of the total variance in reward sensitivity and 5% of the total variance in the learning rate scores.

## Discussion

Combining a laboratory session and an EMA, the current study evaluated whether daily perceived and objective social isolation were associated with anhedonic behavior assessed by performance on the PRT, which provides an objective measure of the ability to adapt behavior as a function of reinforcement history.

The hypothesis of an association between social isolation and dysregulation in motivated behavior was driven by existing evidence of positive cross-sectional and longitudinal associations between social isolation and anhedonic symptoms in human and animal studies (8,38) and of perceived social support as a protective factor for anhedonia and depressive symptoms in general (12,41).

Unexpectedly, momentary levels of perceived and objective social isolation, assessed over 4 consecutive days, were not significantly related to changes in response bias, which is the systematic preference in identifying the more frequently rewarded stimulus across blocks. However, the application of a trial-by-trial computational modeling analysis to PRT choice data allowed us to distinguish 2 critical parameters that underlie such systematic preference, namely reward sensitivity and learning rate. Previous research that attempted to disentangle single critical parameters in the construct of anhedonia described reward sensitivity as the immediate hedonic impact of rewards (or the behavioral equivalent of consummatory pleasure) and learning rate as the participant’s ability to learn from reward feedback, which is shaped by prediction error mechanisms (26).

In the current study, the daily duration of social interactions was negatively associated with reward sensitivity: thus, lesser time spent interacting with others (i.e., higher social isolation) was unexpectedly associated with higher responsivity to rewarding stimuli. Conversely, the duration of daily social interactions was a positive predictor of learning rate, meaning that higher social isolation was associated with an impaired ability to learn from reward feedback, specifically a reduced influence of a given reward on successive behavioral choice.

This conflicting pattern of results is not new in the scientific literature on the effects of social isolation. Behaviorally, the increased reward responsiveness that we found in association with high social isolation resembles the effects of the construct of social craving, which was recently developed by Tomova *et al.* to describe the urge and desire for social interactions that seems to develop following forced isolation in humans and animals (42,43). Conversely, the association of less frequent social interactions with lower reward learning may suggest an impaired ability to learn from reward feedback that may reflect a cognitive adaptation to reduced social stimuli. This could indicate a diminished reinforcement learning capability, possibly due to a generalized decrease in motivation or engagement stemming from prolonged social isolation. This closely resembles what could be termed social despair. Although not explicitly defined as such, this pattern has been observed in both clinical (44) and preclinical studies, particularly during extended or chronic periods of isolation (45). Chronic isolates appear to exhibit a series of profound interpersonal deficits, involving cognitive, affective, and behavioral aspects, linked to reduced motivation and impaired reward learning. In rodents, this is reflected in increased immobility and reduced swimming and climbing behaviors, diminished sucrose preference, and impaired spatial memory and learning (46, 47, 48). Compared with transiently lonely humans, chronically lonely individuals make more self-derogatory internal and stable attributions, seem to prefer passive coping strategies, expect social failure, and fail to seek solutions (49,50).

The opposite associations of social isolation with rewarding sensitivity and learning rate is not surprising if the different neurobiological mechanisms that underpin each parameter are considered. Previous investigations primarily related reward sensitivity and consummatory pleasure to the opioidergic system; specifically, the hedonic impact and liking facet of motivation have been linked to μ opioid signaling in regions such as the shell of the nucleus accumbens (51). On the other hand, preclinical and clinical studies have shown that reward prediction error is primarily tracked by phasic firing of dopaminergic neurons in the midbrain (52, 53, 54). Coherently, the application of the computational model on PRT choice data by Pizzagalli *et al.* showed that administration of a low dose of pramipexole (a dopaminergic agonist acting as an antagonist at low doses) reduced learning rate but not sensitivity (27,55). Thus, it is plausible that the obtained opposite associations between social isolation and the 2 computational factors map onto different and partially dissociable neurobiological mechanisms.

A large body of clinical and preclinical literature points to a role of dopaminergic and opioidergic neuromodulation in regulating social interactions and bonding formation, as well as in social deprivation or social distress (45,56,57). According to preclinical evidence, the endogenous opioid system not only regulates physical pain but also social distress and plays a role in the attribution of value to social interactions (58,59). Coherently, a positron emission tomography study conducted with healthy humans found increased activation in the μ opioid receptor system in the ventral striatum, midline thalamus, amygdala, and periaqueductal gray in individuals exposed to social rejections (60). When comparing individuals with a diagnosis of major depressive disorder to healthy control individuals, however, the same authors found reduced endogenous opioid release during social rejection in brain regions implicated in stress and reward processing (61).

Social isolation, on the other hand, appears to be more specifically related to dopaminergic transmission; in rodents, brief periods of social isolation enhance motivation to seek contact and social interactions with conspecifics, a behavior underpinned by an increase in excitatory inputs to midbrain dopaminergic neurons of the dorsal raphe (42). Again, when chronic isolation is considered, opposite patterns of depressive-like behaviors (increased immobility and despair and reduced sucrose preference indicating impaired reward sensitivity and anhedonia) have been described (48). Consistently, encouraging results linking loneliness (perceived isolation) to weaker activation in reward-related brain regions during exposure to socially rewarding stimuli (vs. objects) has emerged in humans (62). However, these results were not replicated by D’Agostino *et al.* in a similar functional magnetic resonance imaging paradigm (63). To summarize, social isolation and social distress may be modulated by the dopaminergic and opioidergic systems, which in turn may have a preferential impact on reward learning and sensitivity, respectively.

Importantly, most of the reviewed clinical literature concerns loneliness and thus the subjective feeling of isolation involving concepts of the quality of and satisfaction with interactions rather than objective social disconnectedness and lack of relationships per se. According to a large body of research, loneliness represents a greater threat to mental health than physical isolation and seems to be implicated in depression, suicide, alcohol and substance use, poor sleep habits, and dementia (8,9). This is notable considering the current lack of association between self-reported levels of loneliness as assessed by the UCLA Loneliness Scale and reward learning or sensitivity. However, it should be noted that scores on the UCLA Loneliness Scale were negatively associated with the valence of social interaction but not with its daily duration, which confirms the potential of this scale to primarily assess perceived rather than objective social deprivation. Although the literature linking loneliness assessed by the UCLA Loneliness Scale and momentary assessment of social isolation is not extensive, an increasing interest in the issue has evolved, especially during periods of lockdowns and restrictions of public life. As expected, such studies have positively linked daily loneliness to negative subjective states (64) but have failed to find statistically significant associations between quantity and duration of social conversations and scores on the UCLA Loneliness Scale (65).

The unexpected current lack of association between perceived isolation (scores on the UCLA Loneliness Scale) and the components of reward sensitivity and learning may be well explained by the fact that the study was conducted during times when pandemic-related social distancing protocols were in effect. When considering both objective and subjective social isolation, the determinants of objective isolation (living alone, having a small social network, etc.) are considered to be among the most powerful predictors of loneliness (66,67). It is plausible that when objective social disconnectedness reaches an extreme, as in the situation of the severe social restrictions imposed worldwide in the past few years, its effects outweigh the effects of subjective perception of isolation.

The main limitation of the current research is its correlational design, which does not allow for conclusions about causal relationships between the investigated variables (full limitations are detailed in Supplemental Section S9). Although far from unraveling the complex relationships between social isolation and the multifaceted construct of anhedonia, the current study provides preliminary insights into impaired motivational functioning in circumstances of higher daily objective social isolation. Importantly, the use of computational modeling allowed us to add a level of complexity to these well-known associations. Given the involvement of different neurobiological pathways in learning rate and reward sensitivity, replication of the current results may provide meaningful insights for pharmacological and psychotherapeutic early interventions. Because anhedonia is one of the main detrimental mental health effects of the COVID-19 outbreak and its related lifestyle changes and social restrictions (68, 69, 70, 71), understanding the pathways through which forced social isolation influences the specific components of reward learning and sensitivity is important. It is possible that different durations of social isolation distinctively influence each component of reward processing. For example, we speculate that while reward learning deteriorates progressively in response to longer periods of deprivation, sensitivity to reward is enhanced as a coping strategy or compensatory mechanism. Future studies may investigate this hypothesis in humans by administering the PRT during acute mandatory isolation (72) and/or in chronically isolated individuals. In the future, researchers could build on current findings by including a larger and more diverse group of participants in terms of age, sex, and ethnicity to examine whether these variables moderate the observed outcomes.

## Supplementary Material

Key Resources Table
