# Supplementary material for: Daily Social Isolation Maps Onto Distinctive Features of Anhedonic Behavior: A Combined Ecological and Computational Investigation
Source: Biol Psychiatry Glob Open Sci. 2024 Jul 31;4(6):100369. doi: 10.1016/j.bpsgos.2024.100369 (PMC11400617; doi:10.1016/j.bpsgos.2024.100369)

SUPPLEMENTARY INFORMATION

Daily Social Isolation Maps Onto Distinctive Features of Anhedonic Behavior: A Combined Ecological and Computational Investigation

Gigli *et al.*

S1. Exclusionary criteria

Exclusionary criteria were (a) history or presence of serious medical conditions; (b) self-reported formal diagnosis of psychiatric disorder or problematic substance use; (c) neurological disorders; (d) use of drugs/medications; and (e) pregnancy or breast-feeding. Participants were informed that they could win up to 20 euros for their participation.

S2. Revised UCLA Loneliness scale

Examples of items are "How often do you feel that you lack companionship?" or "How often do you feel outgoing and friendly?", and answers are provided on a Likert scale from 0 = Never to 4 = Often. The total score ranges from 20 to 80.

S3. Questions regarding the COVID-19 situation

The following questions about individuals' lifestyle, social isolation (objective or perceived) and recent changes in habits due to the COVID-19 outbreak and subsequent restrictions were administered:

1. Do you live alone?
2. If you do not live alone, with whom do you live?
3. Has the spread of the virus and the restrictions forced you to spend a lot of time alone?
4. Has the spread of the virus and the restrictions significantly impacted your lifestyle and led to substantial changes?
5. Have you ever been in isolation because you contracted COVID-19 or came into contact with someone who tested positive?
6. In the past 6 months, have you been in isolation because you contracted COVID-19 or came into contact with someone who tested positive?
7. Are you currently in isolation?

S4. Probabilistic Reward Task

The PRT was administered in the laboratory via E-Prime (version 3.0). As depicted in Figure S1, the task involves 300 trials, divided into 3 blocks separated by 30-sec breaks. In each trial, participants saw a cross for 1000-1400 ms in the center of the screen, then a cartoon face without the mouth for

500 ms. After that, a mouth appeared for 100 ms. The mouth could be short (10.00 mm) or long (11.00 mm). Next, the cartoon face without the mouth appeared again for an additional 1500 ms. The difference between the short and long mouths was small (just 1 mm), making it difficult to distinguish them (22). Participants were asked to decide if the mouth was short or long by pressing a button ("v" or "m") on the keyboard, counterbalanced across participants. Importantly, not every accurate answer receives a reward. Moreover, and unbeknownst to participants, correct identification of one of the mouth lengths (defined as the "rich stimulus") was rewarded three times more frequently compared to the other ("lean stimulus"). Within each block, the short and long stimuli were presented equally often in a pseudorandomized sequence with the constraint that the same stimulus was not presented more than three times consecutively. For each block, reward feedback ("Correct!! You won 20 cents") was presented after 40 correct responses for 1500 ms after the participant's choice and was followed by 250ms of blank screen. During each block, correct identifications of the rich stimulus received reward feedback for 30 times while correct identifications of the lean one were followed by the positive feedback only 10 times. A controlled reinforcer procedure was used to provide reward feedback according to a pseudorandom schedule that determined which specific trials were to be rewarded for correct choices. If a participant failed to make a correct identification for a trial in which feedback was scheduled, reward feedback was delayed until the next correct response of the same stimulus type (rich or lean). When the reward was not given because the participant was inaccurate (or accurate, but no feedback was scheduled) a blank screen was displayed for 1750 ms. The total task duration was approximately 24 minutes. Participants were informed that they could potentially win up to €20; however, the actual reward was fixed and not contingent on their performance. Therefore, at the conclusion of the task, all participants received a monetary reward of €20.

Response Bias (*log b*) was calculated as:

$$\log b = \frac{1}{2} log \left[\frac{(RICHcorrect + 0.5) \times (LEANincorrect + 0.5)}{(RICHincorrect + 0.5) \times (LEANcorrect + 0.5)}\right],$$

In addition, discriminability (*log d*) was computed as a control measure of participants' ability to discriminate between the two stimuli. Discriminability reflects task difficulty and was calculated as:

$$\log d = \frac{1}{2} lod \left[\frac{(RICHcorrect + 0.5) \times (LEANcorrect + 0.5)}{(RICHincorrect + 0.5) \times (LEANincorrect + 0.5)}\right],$$

0.5 was added to each variable in order to make the calculation of the Response Bias and discriminability possible in cases in which one of the raw cells was equal to 0.

Following previous studies, secondary analyses evaluating accuracy and reaction times (RT) were performed to assess overall task performance. Overall, trials with RTs less than 150 ms or longer than 1500 ms were excluded to remove outliers; then, remaining trials with RTs (following natural log transformation) falling outside the mean 3 ± SD were considered as additional outliers and excluded.

S5. Computation Modeling of PRT

The 'Stimulus-Action' model adopts the standard Rescorla-Wagner premise. Both stimuli were assumed to be totally distinct, and rewards were associated with separate stimulus-action pairs. The 'Action' model proposed that participants only learned the value of their actions when forming expectations, independently of the stimuli. The third model, 'Belief', assumed that participants are uncertain of which stimulus was actually presented on each trial and, thus, the rewards were associated with a combination of two uncertainty-weighted stimulus-action associations. Finally, the 'Punishment' model examined whether trials in which no reward was delivered were treated as aversive losses.

S6. Computation of Learning Rate and Reward Sensitivity in the transformed space

Learning rate is not constrained to the range of 0–1 and reward sensitivity is not constrained to the range of 0 to +inf. Instead, both parameters are unconstrained from –inf to +inf, but larger values still indicate greater learning rate and reward sensitivity. Assuming ε is the actual learning rate with a constraint from 0 to 1, we have presented learning rate in the transformed space as $\log\frac{\varepsilon}{1-\varepsilon}$, which is unconstrained from -inf to +inf. Similarly, assuming ρ is the actual reward sensitivity with a constraint from 0 to +inf, we have presented reward sensitivity in the log-transformed space as $\log\rho$, which is unconstrained from -inf to +inf.

The role of reward sensitivity and learning rate in achieving optimal performance in the task has been explored. Optimal performance was defined as a 3:1 response ratio of rich to lean choices, reflecting the reward probabilities in the task setup (rich trials rewarded at 60% and lean trials at 20%). This ratio is indicative of optimal decision-making, where choice allocation proportionally matches the reward probabilities – a principle derived from the probabilistic matching law.

For the simulations, we varied RS and LR around the mean values observed in our sample data. Specifically, RS and LR were varied by ±1 and ±2 standard deviations from these means. This approach was chosen to reflect a range of behavior that spans from typical to extreme within our observed participant data, thus providing insights into how deviations from the mean affect task performance.

We generated 500 surrogate datasets for each of the 25 RS-LR combinations, with each dataset consisting of 300 trials. We analyzed these datasets using a sliding window of 50 trials to monitor the evolution of the rich:lean response ratio. The results highlighted a nuanced dynamic (Figure S3). For example, if you look at the third row, when learning rate is kept constant at the mean, excessively high RS led participants to disproportionately favour the rich option, deviating from the optimal 3:1 ratio (indicated by the red horizontal line in the plots). Conversely, too low RS resulted in insufficient responsiveness to rewards, failing to leverage the richer option adequately. Regarding LR, if you look at the third column, when RS is kept constant at the mean, high values of LR led to overfitting to recent outcomes, causing fluctuations and instability in choice patterns where the rich:lean response ratio is close to 1. On the other hand, low values of LR hindered timely adaptation to the task's reward contingencies.

These findings illustrate the importance of balancing RS and LR to achieve a response ratio that aligns with the optimal strategy defined by the reward probabilities of the task.

S7. Outlier management

A single outlier at the level of enduring individual differences (i.e., between-persons level) was identified as potentially influential data point using standardized scores >|3| for univariate outliers. Multivariate outliers were identified based on their multivariate Mahalanobis distance (for $p < .01$). While no multivariate outliers were detected, a potentially influential subject was identified for Valence. Removing this subject from the analyses did not change the substantive results, and differences between the zero-order correlations calculated on the final sample and those excluding the univariate outlier did not exceed |.02|.

S8. Ecological Momentary Assessment data analysis

EMA observations (Level 1 or within-person level) were nested within individuals (Level 2, or between-person level). While loneliness and ΔResponse Bias (as well as its subcomponents, reward

sensitivity and learning rate) represents between-person variables by design (i.e., they were measured once), EMA measures represent within-person variables. In both Model 1 and Model 2, the between-person (stable) components of valence and duration of daily social interaction were modeled at the between-person level (i.e., they were centered at their latent means), and their within-person components were specified as unstructured at Level 1 of analysis (i.e., the within-person level was fully saturated).

Participants with < 30% of valid assessments on the EMA measures were not retained for this analysis; however, findings are the same in terms of effect sizes, statistical, and practical significance when a more stringent criterion (<25%) is adopted.

S9. Limitations of the study

The study relies on self-reported measures using a convenience sample, and data were collected in a single national context. Moreover, it is difficult to completely disentangle objective and perceived social isolation in an unselected sample in which the mismatch between actual and perceived social contacts is expected to be lower compared to what is usually seen in clinical populations (1). Notwithstanding, thanks to the use of an EMA design to capture ongoing daily levels of interactions, distinctive patterns of results for actual and perceived social isolation were found. However, the EMA design does not include inquiries about participants' modes of communication, specifically, whether interactions occurred in person or online. This consideration should have been taken into account, as some research suggests that online interactions may detrimentally affect the sense of social bonding, particularly when employed to alleviate social disconnection (2,3). The existing literature is inconsistent on this topic; while several studies support the compensatory theory of technology use in terms of mental well-being and reduced loneliness during the pandemic (4,5), others demonstrate no correlation between the frequency of virtual social interaction and perceived isolation (6). The last limitation is indeed the fact that the present study was conducted during COVID-19 social restrictions (7,8). It has to be noted, however, that studies investigating the role of social relationships during the pandemic showed how perceived social support and social network size may act as a buffer against COVID-related physical and mental health concerns, fatigue and general psychological distress (9,10).

References

1. Wang J, Mann F, Lloyd-Evans B, Ma R, Johnson S (2018): Associations between loneliness and perceived social support and outcomes of mental health problems: a systematic review. *BMC Psychiatry* 18: 156.
2. Geirdal AØ, Ruffolo M, Leung J, Thygesen H, Price D, Bonsaksen T, *et al.*, (2021): Mental health, quality of life, wellbeing, loneliness and use of social media in a time of social distancing during the COVID-19 outbreak. A cross-country comparative study. *J Ment Health* 30: 148–155. https://doi.org/10.1080/09638237.2021.1875413.
3. Bonsaksen T, Ruffolo M, Price D, Leung J, Thygesen H, Lamph G, *et al.*, (2023): Associations between social media use and loneliness in a cross-national population: Do motives for social media use matter? *Health Psychol Behav Med* 11: 2158089. https://doi.org/10.1080/21642850.2022.2158089.
4. Sahi RS, Schwyck ME, Parkinson C, Eisenberger NI (2021): Having more virtual interaction partners during COVID-19 physical distancing measures may benefit mental health. *Sci Rep* 11: 18273. https://doi.org/10.1038/s41598-021-97421-1.
5. Brown G, Greenfield PM (2021): Staying connected during stay-at-home: Communication with family and friends and its association with well-being. *Hum Behav and Emerg* 3: 147–156. https://doi.org/10.1002/hbe2.246.
6. Towner E, Tomova L, Ladensack D, Chu K, Callaghan B (2022): Virtual social interaction and loneliness among emerging adults amid the COVID-19 pandemic. *Curr Res Ecol Soc Psychol* 3: 100058. https://doi.org/10.1016/j.cresp.2022.100058.
7. Bzdok D, Dunbar RIM (2022): Social isolation and the brain in the pandemic era. *Nat Hum Behav* 6: 1333–1343.
8. Forbes PAG, Pronizius E, Feneberg AC, Nater UM, Piperno G, Silani G, *et al.* (2023): The effects of social interactions on momentary stress and mood during COVID -19 lockdowns. *British J Health Psychol* 28: 306–319.
9. Nitschke JP, Forbes PAG, Ali N, Cutler J, Apps MAJ, Lockwood PL, Lamm C (2021): Resilience during uncertainty? Greater social connectedness during COVID-19 lockdown is associated with reduced distress and fatigue. *British J Health Psychol* 26: 553–569.
10. Szkody E, Stearns M, Stanhope L, McKinney C (2021): Stress-Buffering Role of Social Support during COVID-19. *Fam Process* 60: 1002–1015.

Figure S1. Illustration of the Probabilistic Reward Task (A) and graphical representation of the current results in terms of Response Bias (B), Accuracy (C), Discriminability (D), and Reaction Times (E).

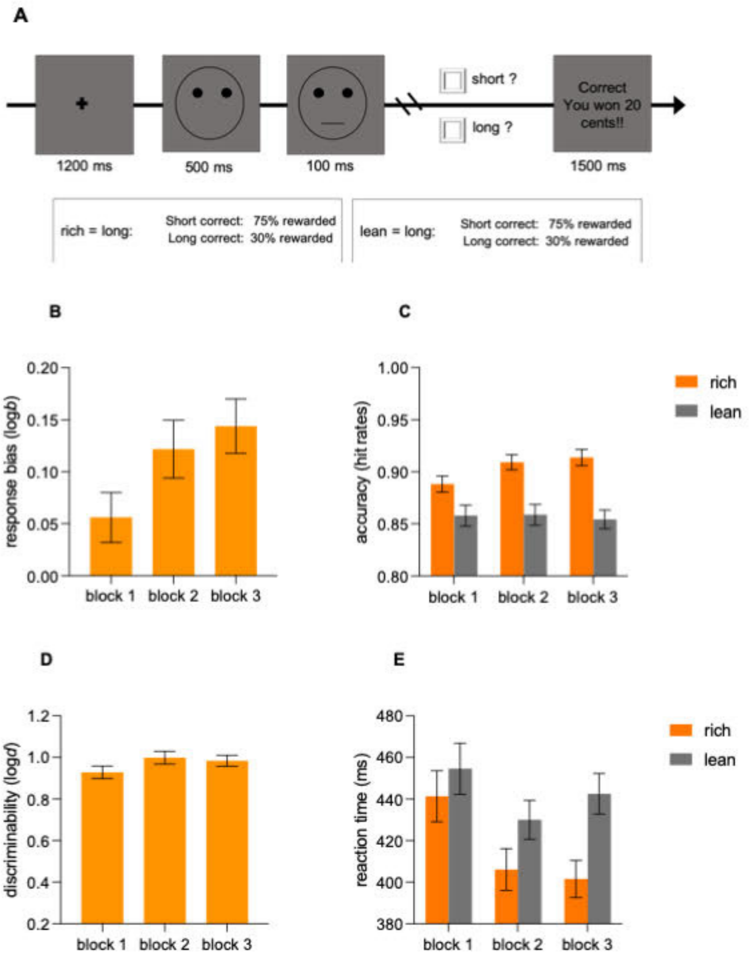

Figure S2. Completely Standardized Estimates from the EMA Model 1.

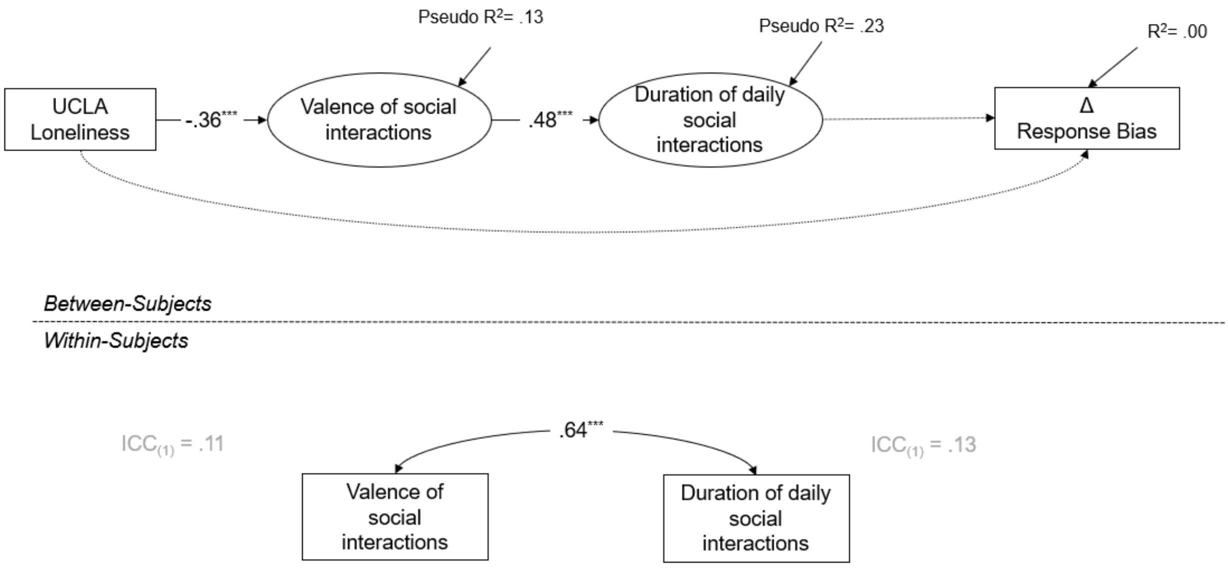

$SB\chi^2_{(df=1)} = .11$, $p = .73$, RMSEA = 0; CFI = 1.00; TLI = 1.00; $SRMR_{within} = .000$; $SRMR_{between} = .008$

Note. RMSEA = Root Mean Square Error of Approximation; CFI = Comparative Fit Index; TLI = Tucker-Lewis Index; SRMR = Standardized Root Mean Square Residual. Dotted lines represent non statistically significant paths. One-headed arrows represent direct effects, while double-headed arrows represent covariances. Squares indicated observed variables, while circles represent latent (between-subject level) components of EMA measures. **p* < .01, ***p* < .01, ****p* < .001.

Figure S3. Role of reward sensitivity (RS) and learning rate (LR) in achieving optimal performance in the PRT.

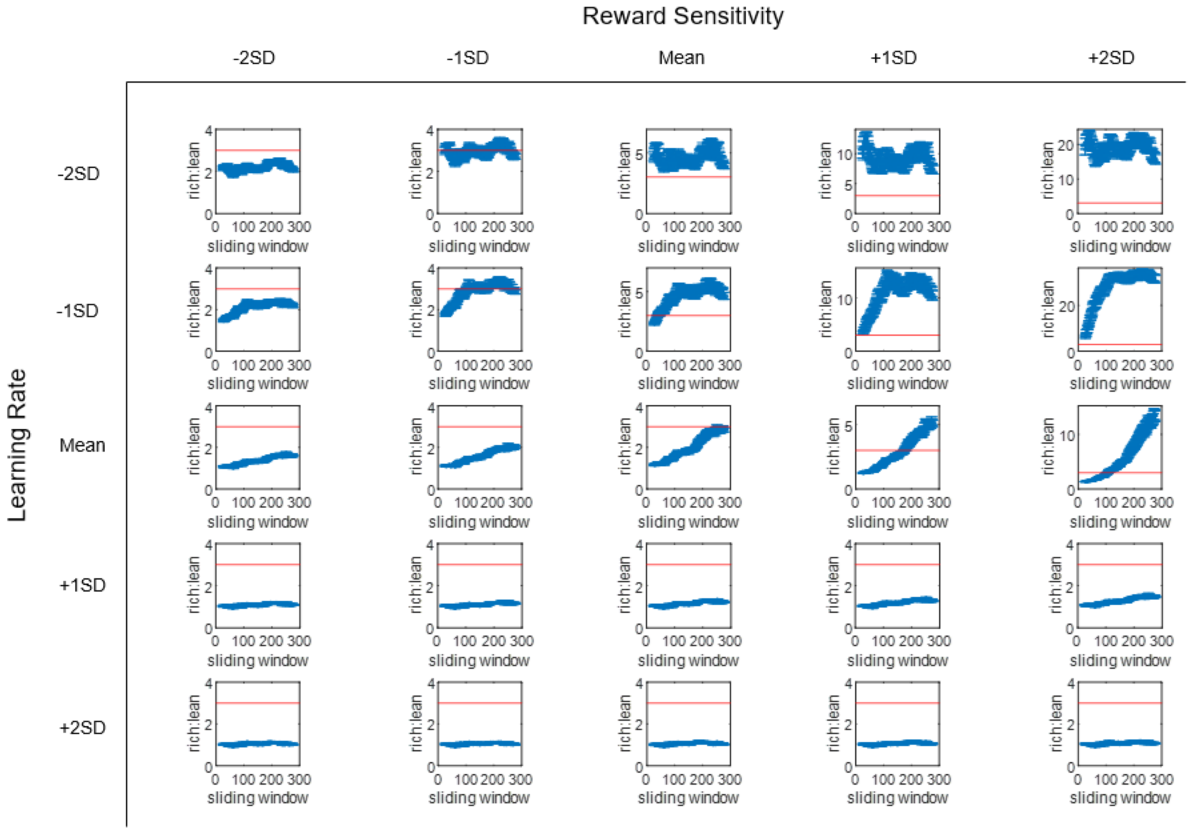

Supplement: Supplement [file mmc2.pdf]
